# Retinal thickness profiles in patients with Behçet uveitis during remission

**DOI:** 10.5935/0004-2749.2021-0174

**Published:** 2022-09-06

**Authors:** Idil Farisogullari, Hilal Eser-Ozturk

**Affiliations:** 1 Ophthalmology Clinic, Akyurt State Hospital, Ankara, Turkey; 2 Department of Ophthalmology, Ondokuz Mayıs University Samsun, Turkey

**Keywords:** Behçet disease, Behçet uveitis, Intraretinal layers, Optical coherence tomography, Retinal segmentation, Doença de Behçet, Uveíte de Behçet, Camadas intrarretinianas, Tomografia de coerência óptica, Segmentação da retina

## Abstract

**Purpose:**

The purpose of this study was to evaluate the intraretinal layer thickness in the macular region and its correlation with the duration of uveitis and visual acuity in patients with Behçet uveitis.

**Methods:**

In this cross-sectional study, we included 93 eyes of 57 patients with Behçet uveitis and 100 eyes of 50 healthy individuals admitted to a tertiary center from January to September 2017. We performed macular measurements in all subjects via spectral domain-optical coherence tomography (SD-OCT) and divided the retina into layers using automated segmentation software on the SD-OCT device. We then compared layer thicknesses between the patient and control groups and evaluated the correlation between OCT parameters and the duration of uveitis and visual acuity in the patient group.

**Results:**

Our records show a mean age of 37.9 ± 10.8 (18-64) years and 37.7 ± 12.2 (21-61) years in the patient and control groups (p=0.821), respectively. Meanwhile, data reveal a mean duration of uveitis of 6.9 ± 4.7 (1-20) years. We found a reduction in the total outer layer thickness in the patient group (p<0.001). However, we did not find a statistically significant difference in the inner retinal layers except in the inner nuclear layer. The duration of uveitis negatively correlated with the outer retinal layer’s thickness (correlation coefficient = -0.250). On the other hand, visual acuity positively correlated with the central macular, the total inner layer, and the outer retinal layer thicknesses (correlation coefficients: 0.194, 0.154, and 0.364, respectively). However, the inner nuclear layer negatively correlated with visual acuity.

**Conclusions:**

Using retinal segmentation via SD-OCT for follow-ups can help estimate visual loss in patients with Behçet uveitis, which can cause significant changes in intraretinal layers in the macular region.

## Abstract (pt)

**Objetivo:**

Avaliar a espessura das camadas intraretinianas na região macular e sua relação com a duração da uveíte e acuidade visual em pacientes com uveíte de Behçet.

**Métodos:**

Este estudo transversal incluiu 93 olhos de 57 pacientes com uveíte de Behçet e 100 olhos de 50 indivíduos saudáveis que foram admitidos em um hospital terciário entre janeiro de 2017 e setembro de 2017. As medições maculares foram realizadas com tomografia de coerência óptica de domínio espectral (SD-OCT) em todos os pacientes. A retina foi dividida em camadas usando software de segmentação automatizado no dispositivo SD-OCT. As espessuras da camada foram comparadas entre os pacientes e os grupos controle. No grupo de pacientes, foi avaliada a correlação entre os parâmetros obtidos na OCT e a duração da uveíte e acuidade visual.

**Resultados:**

A média de idade foi de 37,9 ± 10,8 (18-64) no grupo de pacientes e 37,7 ± 12,2 (21-61) no grupo controle (p=0,821). A duração média da uveíte foi de 6,9 ± 4,7 (1-20) anos. A espessura total das camadas externas no grupo de pacientes foi reduzida (p<0,001). Uma diferença estatisticamente significativa não foi encontrada nas camadas internas da retina, exceto na camada nuclear interna. Uma correlação negativa foi detectada entre a duração da uveíte e a espessura da camada externa da retina (coeficiente de correlação = -0,250). Uma correlação positiva significativa foi detectada entre a acuidade visual e a espessura macular central bem como a espessura total das camadas internas e externas da retina (coeficientes de correlação 0,194; 0,154 e 0,364, respectivamente). A camada nuclear interna foi negativamente correlacionada com a acuidade visual.

**Conclusões:**

A uveíte de Behçet pode causar alterações significativas nas camadas intraretinianas na região macular. A segmentação da retina com SD-OCT pode ser útil para acompanhamentos e para estimar a perda visual em pacientes com uveíte de Behçet.

## INTRODUCTION

Behçet disease, a recurring multisystem inflammatory disorder, affects the eyes, skin, and joints, as well as the gastrointestinal, cardiovascular, and neurologic systems. It is significantly common in countries along the ancient “Silk Road”^(1)^, with the highest reported prevalence rate in Turkey^(2)^. Because of a lack of specific diagnostic tests, diagnosing the disease is based on clinical findings, and the most commonly used criteria were developed by the International Study Group (ISG)^(3)^. Behçet uveitis (BU) is characterized by bilateral non-granulomatous uveitis and retinal vasculitis. Ocular involvement occurs in around two third of the patients. The active stage of BU may also exhibit diffuse vitritis, superficial retinal infiltrates, retinal vein occlusions, retinal hemorrhages, inflammatory sheathing of retinal vessels, optic nerve involvements, and retinal neovas-cularization. In addition, recurrent posterior segment episodes may lead to permanent fundus changes such as optic atrophy, diffuse retinal atrophy, and atrophic maculopathy, significantly impairing visual acuity^(4,5,6,7)^.

Although fundus fluorescein angiography is the gold standard technique in the diagnosis and follow-up of BU, OCT is a noninvasive technique for displaying structural changes of the posterior segment in patients with BU^(8)^. Previous studies found that reduced foveal thickness and ellipsoid zone (EZ) impairment correlated with low visual acuity^(9,10,11)^. In addition, Oray et al.^(12)^ observed that retinal infiltrates, which develop during the active inflammation phase of BU and recover without visible scarring, could cause local retinal nerve fiber layer (RNLF) defects. The authors proposed that RNLF defects in the posterior pole indicate previous episodes and may be evaluated as a prognostic factor for visual acuity^(12)^. Meanwhile, Kido et al.^(13)^ reported that previous uveitis episodes could cause outer plexiform layer elevations associated with decreases in retinal thickness and visual acuity. These studies provided important clues that BU could affect different intraretinal layers.

High-resolution spectral domain OCT (SD-OCT) and automated segmentation programs measure each retinal layer in the macular region, making the evaluation of structural pathologies and their relationship to different diseases possible in detail^(14,15,16,17)^.

This study aimed to investigate the changes in the intraretinal layer thickness in the macular region of patients with BU who are clinically and angiographically in remission. We also evaluated whether altered thickness correlated with visual acuity and the duration of uveitis.

## METHODS

We conducted this cross-sectional study in the Ophthalmology Department, Ondokuz Mayis University between January and September 2017. We included 64 patients with BU, being followed up at the Uvea-Behçet clinic, and 50 healthy individuals, admitted to the outpatient clinic for refractive errors, in the study. The patient group comprised patients who met the International Study Group’s criteria for Behçet disease who had posterior segment involvement^(3)^. We obtained ethics committee approval from Ondokuz Mayis University Medical School (OMU KAEK 2017/344) for the study, following the principles of the Declaration of Helsinki.

Inclusion criteria included the following: (1) older than 18 years, (2) with posterior segment involvement of BU, (3) with inactive phase for ocular involvement, and (4) with a spherical equivalent refractive error between −3 and +5 diopters. We defined the inactive phase as the absence of inflammation, such as iridocyclitis, hypopyon, vitritis, vasculitis, retinitis, and optic disc and macular edema, and the absence of vascular and capillary leakage on fluorescein angiography. We excluded patients with macular atrophy from previous uveitis attacks. We also excluded patients with comorbid conditions that could influence the macula and optic nerve and primary ocular pathologies, including glaucoma, age-related macular degeneration, and hereditary retinopathy. We then recorded age, gender, duration of uveitis, and received systemic therapies.

We had all patients and control subjects undergo a complete ophthalmologic examination, including refractive error measurement using an automated refractometry device (KR 8100 P; Topcon, Tokyo, Japan), best corrected visual acuity (BCVA) measurement using the Snellen chart, intraocular pressure (IOP) measurement using Goldman applanation tonometry, slit-lamp biomicroscopy, and dilated fundus examination. We obtained macular OCT images using a Heidelberg Spectralis (Heidelberg Engineering, Heidelberg, Germany) device. We then used the 25-line raster SD-OCT acquisition pattern to assess the macular anatomy. We processed the obtained images using the device’s automated segmentation software and divided the retina into layers. The device automatically measured the layer thicknesses. We also evaluated the integrity of EZ (formerly the inner/outer segment of photoreceptors [IS/OS]), interdigitation zone (IZ; formerly the cone outer segment tips [COST]), and external limiting membrane (ELM) in the patient group.

**Figure 1:**
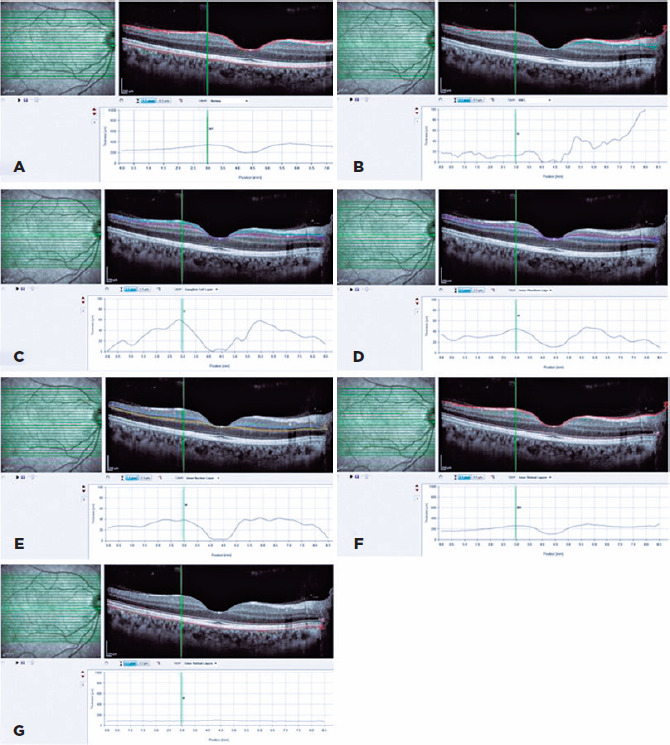
The retinal layer thickness that were calculated automatically by spectral domain-optical coherence tomography (SD-OCT) evaluated in this study: (A) central macular thickness (CMT), (B) macular retinal nerve fiber layer (RNLF), (C) ganglion cell layer, (D) inner plexiform layer, (E) inner nuclear layer, (F) total inner layer thickness, and (G) total outer layer thickness were evaluated.

This study used the data on (a) central macular thickness (CMT), (b) macular retinal nerve fiber layer (RNFL), (c) ganglion cell layer, (d) inner plexiform layer, (e) inner nuclear layer, (f) total inner layer thickness, and (g) total outer layer thickness. The total inner layer thickness was defined as the distance between the internal limiting membrane and the inner plexiform layer. We defined the total outer layer thickness as the distance between the outer plexiform layer and the Bruch membrane (Figure 1). Considering these data, we then compared the patient and control groups and performed a correlation analysis between the changes in these layers and the duration of uveitis and visual acuity. We constructed linear regression models to examine the effects of each independent variable on visual acuity. In addition, we divided the patients into four groups according to the received treatment: conventional therapy (azathioprine and/or cyclosporine) in the first group, interferon in the second group, and anti-TNF agents in the third group. Patients who did not receive systemic immunosuppression were in group 4. We compared the thicknesses of the intraretinal layers between the groups.

### Statistical analysis

We analyzed data using the IBM SPSS (version 23, SPSS Inc., Chicago, IL, USA) program and tested the normal distribution via Shapiro-Wilk test. We tested independent samples using *t*-test to compare data that showed normal distribution. On the other hand, we used Mann-Whitney U and Kruskal-Wallis tests to compare data that did not show normal distribution. Meanwhile, we used Spearman correlation to evaluate the association between variables and the χ^2^ test to analyze categorical variables. We expressed the data that showed normal distribution as mean ± standard deviation, whereas we expressed the data that did not show normal distribution as the median (min-max). We expressed qualitative data as frequency (percent). For the linear regression model, we assessed the model fit using F-test and evaluated the independence of residuals using the Durbin-Watson test. We considered a *p*-value <.05 as significant at 95% confidence interval.

## RESULTS

We excluded from the study seven of the 64 patients because of glaucoma, macular atrophy, and/or cystoid macular edema (CME). We evaluated data on 93 eyes and 100 eyes from 57 patients and 50 healthy individuals, respectively, who were age- and gender-matched. Table 1 presents the distribution of age, gender, duration of uveitis, spherical equivalent of refractive error, and BCVA.

**Table 1:** Characteristics of the patient and control groups

	Patient group	Control group	p-value
Patient n/eye n	57/93	50/100	
Mean age (y) ± SD	37.9 ± 10.8	37.7 ± 12.2	0.821
Gender F/M	40/17	34/16	0.973
Mean duration of uveitis (y) ± SD	6.9 ± 4.7	N/A	
Median spherical equivalent (min to max)	−.25 (−3.00 to +3.00)	−.5 (−3.00 to +4.00)	0.085
Median BCVA (min–max)	0.9 (0.01-1.0)	1.0 (.9-1.0)	**0.000**

BCVA= best corrected visual acuity; F= female; M= male; max= maximum; min= minimum; n= number; N/A= nonapplicable; SD= standard deviation; y= years.

None of the patients presented symptoms of active inflammation or CME. We detected epiretinal membrane (ERM) in 29 (31.2%) eyes. Twenty-seven patients were on conventional immunosuppressants (azathioprine and/or cyclosporine); eight on interferon; and 12 on antitumor necrosis factor alpha (TNF-α) agents (adalimumab or infliximab). Only three patients were on additional oral corticosteroids (<10mg/day). On the other hand, 10 patients were not on any treatment other than colchicine.

Comparing macular segment thickness data between groups, we detected statistically significant thinning in the outer retinal layers of patients with BU (p<0.001). We also found thicker inner nuclear layer in the patient group than in the control group (p=0.003). However, we did not find any significant difference in the thickness of other layers between groups (Table 2).

**Table 2:** Retinal layer thickness obtained via OCT in the patient and control groups

	Patient	Control	p-value
CMT, µam; mean ± SD	261.8 ± 37.3	270.4 ± 25.1	0.106
Macular RNFL, µm; median (min-max)	12 (3-33)	12 (7-24)	0.290
Ganglion cell layer, µm; median (min-max)	15 (5-50)	15 (8-54)	0.928
Inner plexiform layer, µm; median (min-max)	20 (14-43)	20 (14-48)	0.977
Inner nuclear layer, µm; median (min-max)	20 (8-55)	18 (9-51)	**0.003**
Total inner layer thickness, µam; mean ± SD	175.6 ± 35.9	180.9 ± 26.3	0.211
Total outer layer thickness, µm; median (min-max)	87 (74-103)	90 (76-96)	**<0.001**

CMT= central macular thickness; max= maximum; µm= micrometer; min= minimum; OCT= optical coherence tomography; RNFL= retinal nerve fiber layer; SD= standard deviation.

We detected disruptions in EZ, IZ, and ELM in 20 (21.5%), 26 (28%), and 10 (10.8%) eyes in the patient group, respectively.

We analyzed the correlation between macular segment thickness values and the duration of uveitis and visual acuity in the patient group. We detected a decrea se in outer layer thickness as the duration of uveitis prolonged (correlation coefficient = −0.250). The CMT, total inner layer thickness, and total outer layer thickness positively correlated with visual acuity (correlation coefficients: 0.194, 0.154, and 0.364, respectively). However, the inner nuclear layer negatively correlated with visual acuity (Table 3).

**Table 3:** The correlation between the duration of uveitis, visual acuity, and macular segment thickness obtained via OCT

	Duration of uveitis	Visual acuity
CMT (µm)	−0.055 (p=0.696)	0.194* (p=0.007)
Macular RNFL (µm)	0.037 (p=0.794)	−0.017 (p=0.812)
Ganglion cell layer (µm)	0.064 (p=0.648)	0.022 (p=0.758)
Inner plexiform layer (µm)	0.062 (p=0.660)	0.078(p=0.280)
Inner nuclear layer (µm)	0.177 (p=0.206)	−0.186** (p=0.010)
Total inner layer thickness (µm)	−0.024 (p=0.864)	0.154* (p=0.032)
Total outer layer thickness (µm)	−0.250* (p=0.018)	0.364** (p=0.000)

CMT=central macular thickness; µm= micrometer; OCT= optical coherence tomography; RNFL= retinal nerve fiber layer.

*= significant correlation at 5% significance level; **= significant correlation at 1% significance level.

Among these retinal thickness values, we identified the thickness of the inner nuclear layer as the only independent predictor of visual loss on multivariate linear regression analysis. In addition, we identified the disruptions in EZ and IZ as independent predictors of visual loss (Table 4).

**Table 4:** Results of linear regression analysis for detecting independent variables on visual acuity

Predictors	Beta	Sh	t	p-value
CMT (µm)	−0.014	0.010	−1.410	0.160
Inner nuclear layer (µm)	−0.006	0.002	−3.101	**0.002**
Total inner layer thickness (µm)	0.016	0.010	1.554	0.122
Total outer layer thickness (µm)	0.011	0.010	1.117	0.266
EZ disruption	−0.191	0.047	−4.066	**0.000**
IZ disruption	−0.228	0.040	−5.710	**0.000**
ELM disruption	0.001	0.054	0.025	0.980

CMT= central macular thickness; ELM= external limiting membrane; EZ= ellipsoid zone; IZ= interdigitation zone; µm= micrometer. Model (R=0.773, R2=0.598; F=39.090; p=0.000).

After analyzing the retinal layer thickness between treatment groups, we found no statistically significant difference (Table 5).

**Table 5:** Retinal layer thickness obtained via OCT in diferent treatment groups

	Conventional (n=44)	Interferon (n=13)	Anti-TNF (n=18)	No treatment (n=18)	p-value
CMT, µm; median (min-max)	257 (202-313)	266 (188-324)	246 (173-325)	266 (210-307)	0.494
Macular RNFL, µm; median (min-max)	12 (8-22)	12 (8-33)	13 (8-20)	12 (6-19)	0.950
Ganglion cell layer, µm; median (min-max)	15 (7-48)	14 (8-50)	15 (5-30)	13 (8-29)	0.927
Inner plexiform layer, µm; median (min-max)	20 (14-38)	20 (14-43)	21 (14-42)	20 (15-33)	0.949
Inner nuclear layer, µm; median (min-max)	19 (13-42)	17 (11-39)	19 (8-55)	21 (13-33)	0.814
Total inner layer thickness, µm; median (min-max)	172 (119-281)	179 (109-243)	163 (94-250)	177 (128-222)	0.544
Total outer layer thickness, µm; median (min-max)	87 (79-103)	84 (74-103)	86 (74-98)	87 (79-95)	0.543

Anti-TNF= antitumor necrosis factor; CMT= central macular thickness; max= maximum; µm= micrometer; min= minimum; n= number of eyes; OCT= optical coherence tomography; RNFL= retinal nerve fiber layer.

## DISCUSSION

Inadequate treatment of BU, which is characterized by recurrent uveitis episodes and spontaneous resolutions, can lead to smoldering inflammation despite clinical inactivity, as well as complications like macular edema and neovascularization. Achieving angiographic remission is essential to effective treatment, as angio-graphy can detect vascular and capillary leakage or obstructive vasculitis-related nonperfused field in patients with clinically inactive BU. If complete remission is not achieved, recurrent posterior segment episodes might lead to diffuse retinal atrophy, optic atrophy, and/or macular atrophy, which lead to permanent visual loss. Morphologic changes in the retina can be detected via OCT^(4,5,6,8)^.

High-resolution SD-OCT and automated segmentation programs can visualize intraretinal layers in the macular region and measure layer thickness^(14,15,16)^. This study did not find a significant difference when comparing mean CMT values between the patient and control groups. The thickness of macular RNFL, ganglion cell layer, inner plexiform layer, and total inner layer thickness did not differ between the groups. However, we found a thicker inner nuclear layer in the patient group. Cheng et al.^(17)^ also evaluated patients who were in Behçet disease remission and found significant thickening in the inner retinal layers, associating this thickening with incomplete control of inflammation and a subclinical inflammatory effect. The authors reported the RNFL’s greater susceptibility to an inflammatory effect, particularly at the nasal part of the macula, suggesting that RNFL thickness is an indicator of subclinical macular involvement. In addition, previous studies reported that acute inflammation could lead to inner retinal layer thickening ^(18,19)^. In this study, the patient group had thinner total inner layer thickness than the control group, albeit not statistically significant. The reason for the detection of thinning in the total inner retinal layers may be due to the fact that we aim not only clinical but also angiographic remission in clinical practice. Despite the decrease in total inner layer thickness in our study, we found that the patient group had significantly thicker inner nuclear layer. Previous studies had shown an increase in the thickness of the inner nuclear layer, which had the greatest correlation with visual acuity in the presence of ERM^(20,21)^. We thought that the presence of ERM in 29 (31.7%) eyes lead to the thickening in the inner nuclear layer in our study.

On the other hand, Cheng et al.^(17)^ detected a thinning in the outer retinal layers in their study, which was more prominent in the patient group that had more than 3 years of disease duration, compared with the group with a shorter disease duration and the control group. The authors concluded that the thinning correlated with permanent and progressive photoreceptor damage in long-term disease and that progressive photoreceptor damage correlated with recurring macular edema episodes, leading to neurosensory retinal damage due to retinal vessel inflammation. Similarly, our study found a thinning in the outer retinal layer, showing a negative correlation with the duration of uveitis. In other words, thinning in the outer retinal layers become more prominent as the duration of uveitis prolonged.

Researchers investigating the correlation between the decrease in visual acuity and macular thickness revealed different results^(9,10,11,22)^. Takeuchi et al.^(9)^ found that macular thinning occurred as the duration of uveitis prolonged. However, thinning did not correlate with visual acuity. The authors explained the relationship between duration of uveitis and macular thinning by noting that vasculitis may be seen as a hyper-permeable type or occlusive type in BU, with the latter being more common in the late period. Cheng et al.^(17)^ detected thickening in RNFL and inner nuclear layers, suggesting that this increase in thickness correlated with the reduction in visual acuity. This condition could be due to the continuation of subclinical inflammation as reported by the authors. In addition, we found that the inner nuclear layer thickness negatively correlated with visual acuity. We assumed that the presence of ERM increased the thickness of the inner nuclear layer and consequently decreased visual acuity. In this study, we found that the CMT and total inner layer thickness positively correlated with visual acuity. A previous study reported that the CMT could decrease as the duration of uveitis progresses^(9)^. The episodes affecting the areas close to the vision center can cause retinal thinning^(12)^. Therefore, the CMT and total inner layer thickness possibly correlated with visual acuity in patients who were in the inactive period and had prolonged uveitis.

Our study found a positive correlation between thinning in the outer retinal layers and visual acuity. Cheng et al.^(17)^ demonstrated that visual acuity correlated with the thinning of outer retinal layers and EZ impairment, proposing that outer retinal layer thickness reflects the integrity of the photoreceptor layer besides EZ, making it a useful marker for estimating visual acuity. Other studies also demonstrated an association between EZ damage and impaired visual acuity ^(10,11)^. Unoki et al.^(10)^ reported that IS/OS band disruptions, which reflect disorganization of the photoreceptor layer, caused thinning of the macular region, making them a possible marker of irreversible visual loss. On the other hand, Yüksel et al.^(11)^ revealed that IS/OS band integrity and macular thickness correlated with visual acuity; however, in a linear regression analysis, they found that only IS/OS line integrity was an independent variable for visual acuity.

Our study demonstrated the inner nuclear layer as an independent predictor for visual loss in linear regression analysis. The CMT, total inner layer thickness, and total outer layer thickness, which correlated with visual acuity, lost their significance in regression analysis. A previous study found the nasal outer retinal layer, temporal and nasal inner retinal layer, and inferior nerve fiber layer as independent predictors of BCVA ^(17)^. Our study also identified the EZ and IZ disruptions as independent predictors of visual loss, similar to other studies^(11,17)^.

Evaluating whether the treatment received by the patients affected the thickness of intraretinal layers, we found that received treatment did not affect the thickness of the intraretinal layers. This could be due to the fact that patients were in clinical and angiographical remission, regardless of the treatment they received.

The most important limitation of our study is its cross-sectional design and its limited number of patients. Another limitation is the inaccessibility to data before patients’ admission to our hospital, which is a reference center and could determine incomplete detection of the number of episodes. Therefore, we were not able to evaluate the influence of the number of episodes on retinal thicknesses.

In conclusion, although we could not detect a significant difference in the total inner layer thickness in patients with BU compared with controls, we observed a reduction in the total outer layer thickness. Unlike previous studies, we did not detect inner retinal layer thickening, which was suggested as a subclinical inflammation indicator. This may be due to our aggressive treatment approach that targeted both clinical and angiographic remissions. We believe that the thinning of the outer retinal layers as the disease duration progresses may be related to the number of uveitis attacks. As early and aggressive treatment can prevent atrophic changes in the outer retinal layers, the use of retinal segmentation with SD-OCT as a noninvasive method can help in evaluating the response to treatment and estimating visual loss in patients with BU.
